# Sustained-Release Microspheres of Rivoceranib for the Treatment of Subfoveal Choroidal Neovascularization

**DOI:** 10.3390/pharmaceutics13101548

**Published:** 2021-09-24

**Authors:** E Seul Kim, Min Sang Lee, Hayoung Jeong, Su Yeon Lim, Doha Kim, Dahwun Kim, Jaeback Jung, Siyan Lyu, Hee Joo Cho, Dong Min Kim, Wonhee Suh, Ji Hoon Jeong

**Affiliations:** 1 School of Pharmacy, Sungkyunkwan University, Suwon 16419, Korea; seul4146@skku.edu (E.S.K.); lminsa@skku.edu (M.S.L.); kally37@naver.com (S.Y.L.); gnjs0219@naver.com (D.K.); domonde@naver.com (J.J.); lsy2018dd@hotmail.com (S.L.); chohj823@naver.com (H.J.C.); eastjade@g.skku.edu (D.M.K.); 2 Department of Global Innovative Drug, Graduate School of Chung-Ang University, Seoul 06974, Korea; gkdud819@naver.com (H.J.); summerflow02@gmail.com (D.K.); 3 College of Pharmacy, Chung-Ang University, Seoul 06974, Korea

**Keywords:** rivoceranib, drug repositioning, microsphere, subfoveal choroidal neovascularization, macular degeneration

## Abstract

The wet type of age-related macular degeneration (AMD) accompanies the subfoveal choroidal neovascularization (CNV) caused by the abnormal extension or remodeling of blood vessels to the macula and retinal pigment epithelium (RPE). Vascular endothelial growth factor (VEGF) is known to play a crucial role in the pathogenesis of the disease. In this study, we tried to repurpose an investigational anticancer drug, rivoceranib, which is a selective inhibitor of VEGF receptor-2 (VEGFR2), and evaluate the therapeutic potential of the drug for the treatment of wet-type AMD in a laser-induced CNV mouse model using microsphere-based sustained drug release formulations. The PLGA-based rivoceranib microsphere can carry out a sustained delivery of rivoceranib for 50 days. When administered intravitreally, the sustained microsphere formulation of rivoceranib effectively inhibited the formation of subfoveal neovascular lesions in mice.

## 1. Introduction

The eye is a representative organ, exhibiting the fastest signs of aging, and visual problems are generally more noticeable than other age-related disorders [1]. Age-related macular degeneration (AMD) is a leading cause of irreversible visual loss in older adults [2]. The impaired vision and blindness associated with AMD are due to atrophic and neovascular complications [3]. Although the dry or non-neovascular type of AMD is more prevalent than wet or neovascular AMD, wet AMD is often involved with severely reduced vision acuity and vision loss which occurs when the outgrowth of blood vessels in the choroidal region extends underneath the foveal avascular zone (subfoveal choroidal neovascularization (CNV)) [4,5]. Vascular endothelial growth factor (VEGF) is one of the most critical factors for the vascular proliferation and migration of endothelial cells in the neovascularization process [6]. Therefore, blocking the VEGF signaling pathway to prevent CNV has been popularly employed for the treatment of wet AMD [7]. Monoclonal antibodies targeting VEGF receptors (VEGFR) such as bevacizumab and ranibizumab are frequently used for CNV due to their desirable effectiveness and low incidence of serious ocular and systemic adverse events [8]. However, the lack of a proper delivery system and the high cost of antibody therapy have remained a limitation [9].

Corticosteroids are also widely used for ocular disorders involving CNV and macular edema, owing to their anti-inflammatory and anti-angiogenic properties [10]. Intravitreal injectable implants containing corticosteroids such as dexamethasone and fluocinolone acetamide could achieve sustained drug release in vitreous space for an extended period. However, steroid therapy is often associated with adverse events, including cataract formation, ocular hypertension, and glaucoma [11,12,13].

Biodegradable microspheres have been used for the extended release of various active substances including small molecules, polypeptides, and nucleic acids, since they are injectable and biodegradable, so additional surgical procedures for implant removal are not required [14]. In addition, microspheres can be formulated with hydrophobic as well as hydrophilic drugs and deliver the active substances for a longer period compared to nanoparticles [15]. Microspheres containing a small molecular protein kinase C inhibitor (PKC412, Novartis Pharma) and fasudil were developed for CNV and glaucoma, respectively [16,17]. A sustained-release microsphere for an anti-VEGF RNA aptamer was also designed for the treatment of AMD [18].

Rivoceranib is a selective receptor tyrosine kinase inhibitor targeting the intracellular ATP-binding site of VEGFR2. Since VEGFR2 plays a crucial role in VEGF-mediated endothelial cell proliferation, migration, and permeabilization [19], rivoceranib was developed for antiangiogenic cancer therapy, and is in clinical trials for metastatic gastric cancer and metastatic adenoid cystic carcinoma [20]. We hypothesized that rivoceranib, a selective inhibitor of VEGFR tyrosine kinase, could be repositioned as a candidate for an anti-VEGF therapy for CNV. In our previous studies, human albumin-polyethyleneglycol (HSA-PEG) conjugate-based nanoparticles containing rivoceranib effectively reduced retinal vascular leakage and corneal neovascularization by blocking VEGF/VEGFR2 signaling [21,22]. In this study, injectable microsphere dosage forms of rivoceranib were developed for extended drug release in the vitreous space. The potential therapeutic effect of the rivoceranib microspheres for wet AMD was evaluated in a laser-induced CNV animal model.

## 2. Materials and Methods

### 2.1. Materials

Rivoceranib was obtained from HLB Bio (Seoul, Korea). Poly(d,l-lactic-co-glycolic acid) (PLGA) copolymers (Resomer RG502 (50:50, Mw 7000–17,000), Resomer RG502H (50:50, Mw 7000–17,000) and Resomer RG503H (50:50, Mw 24,000–38,000)), polyvinyl alcohol (PVA), and Tween 20 were purchased from Sigma (St. Louis, MO, USA). Methylene chloride, acetonitrile, and methanol were of analytical grade and used without purification (J. T. Baker, Phillipsburg, NJ, USA). All the liquid solutions were sterilized by autoclave or filtration (0.45 µm filter unit, Millipore, Billerica, MA, USA).

### 2.2. Preparation and Characterization of Rivoceranib Microspheres

Microspheres containing rivoceranib were fabricated using an oil-in-water emulsification method. PLGA (360 mg) and rivoceranib (40 mg) were dissolved in methylene chloride/methanol (9:1 *v/v*, 1 mL). The solution was slowly added to 0.5% PVA solution (*w/v*, 300 mL) and emulsified with a high-speed dispersion homogenizer (HG-15D, Daihan Scientific, Seoul, Korea) at either 1500 or 2000 rpm for 2 min. The resulting microspheres were centrifuged at 2000 rpm at 4 °C and the supernatant was removed. The microspheres were resuspended in cold deionized water and washed three times by the centrifugation method. The collected microspheres were freeze-dried at −70 °C and stored at −20 °C until use. The drug loading and encapsulation efficiency of the microspheres were determined using a Waters 626 HPLC pump equipped with a UV detector (Waters, Milford, MA, USA) and C18 reversed-phase column (5 μm particle size; Millipore, Billerica, MA, USA). The mobile phase (acetonitrile and 0.01 M KH_2_PO_4_ in deionized water, 70:30 *v/v*) was delivered at a flow rate of 1 mL/min. The detection wavelength was set at 260 nm. The morphology and size distribution of the rivoceranib microspheres were observed using scanning electron microscopy (SEM, JSM7600F, JEOL, Tokyo, Japan) and dynamic light scattering (Mastersizer 2000, Malvern Panalytical, Malvern, UK).

### 2.3. Differential Scanning Calorimetric (DSC) and Fourier-Transform Infrared (FTIR) Spectral Studies

A mixture of PLGA and rivoceranib (1:1, *w*/*w*) was dissolved in a cosolvent of methylene chloride/methanol (9:1, *v/v*) and the solvent was evaporated to form a film. For DSC analysis, one milligram of the film sample was placed in an aluminum pan and its thermal behavior was monitored by applying heat from 25 °C to 200 °C at a heating rate of 5 °C/min (DSC 6100, Seiko Instruments, Chiba, Japan). For FTIR analysis, the spectral profile of PLGA/rivoceranib film was observed in the range of 400 to 4000 cm^−1^ using FTIR spectrophotometer (IFS66v-S, Bruker, Billerica, MA, USA).

### 2.4. Drug Release Profiles of PLGA/Rivoceranib Microspheres

The PLGA/rivoceranib microspheres (5 mg) were suspended in 1 mL of deionized water. The suspension was transferred to a dialysis bag (MWCO 5000, Specrum, Gardena, CA, USA) and drug release was carried out in a 10 mL release medium (0.1% polysorbate 80 in PBS pH 7.4) at 37 °C. The samples were taken at a predetermined period and the release medium was replenished. The amount of released drug was analyzed using HPLC.

### 2.5. Animals

The animals were cared for in accordance with the Guide for the Care and Use of Laboratory Animals published by the United States National Institutes of Health. The protocols were also approved by the Institutional Animal Care and Use Committee (IACUC) of Chung-Ang University (IACUC number: 201800044, start date: 4 May 2018). Nine- to ten-week-old male C57BL/6 mice were purchased from Orient Co., Ltd. (Seoul, Korea). Mice were housed in microisolator cages on individually ventilated cage racks with ad libitum access to an autoclaved standard rodent diet (LabDiet 5008, Purina, St. Louis, MO, USA) and were kept under a 12:12 h light/dark cycle. Anesthesia was performed via an intraperitoneal injection of ketamine hydrochloride (100 mg/kg body weight) and xylazine hydrochloride (6 mg/kg body weight). The pupils of the anesthetized mice were dilated with topical drops of 1% tropicamide (Santen, Osaka, Japan).

### 2.6. Mouse Model of Laser-Induced Choroidal Neovascularization (CNV)

Immediately after mice were anesthetized and their pupils dilated, experimental CNV lesions (four spots per eye) were created at the 3, 6, 9, and 12 o’clock positions of the posterior pole of the fundus at equal distances from the optic nerve head with the following parameters: 532 nm wavelength, 50 µm diameter, 70 ms duration, and 220 mW intensity (Micron IV image-guided laser system, Phoenix Research Laboratories, Pleasanton, CA, USA). Only mice with cavitation bubbles, which indicated the disruption of Bruch’s membrane, were included in the study. Immediately after CNV induction, the mice were given an intravitreal injection of rivoceranib-loaded PLGA microspheres (10 µg rivoceranib/10% RG502H−2.0k in 1 µL PBS), PLGA microspheres (10% RG502H−2.0k in 1 µL PBS), or PBS (1 µL). Two weeks later, the mice were euthanized for further analysis.

### 2.7. Quantification of Laser-Induced CNV

The eyes were enucleated and fixed in 4% paraformaldehyde solution in PBS. The posterior eyecups comprising the retinal pigmental epithelium, choroid, and sclera were microdissected from the surrounding tissues and prepared as flat mounts. The flat mounts were then stained with Alexa Fluor^®^ 594-conjugated Griffonia simplicifolia isolectin B4 (IB4; 1:100 dilution; Invitrogen, Carlsbad, CA, USA) overnight at 4 °C. Images of CNV lesions were obtained using a fluorescence microscope (Olympus, Tokyo, Japan); the exposure and gain were kept constant for all samples. In each whole-mount image, the numbers of pixels in the CNV areas were measured using ImageJ software (National Institute of Health, Bethesda, MD, USA).

### 2.8. Statistical Analysis

All data are expressed as the mean ± standard error of mean (SEM) of indicated n values. One-way analysis of variance was used to determine the significance of differences between groups. Data were analyzed using the Bonferroni correction for multiple comparisons. Differences were considered significant when *p* < 0.05.

## 3. Results

### 3.1. Preparation and Characterization of Rivoceranib Microspheres

The rivoceranib microspheres were prepared using an oil-in-water emulsification process, in which the manufacturing parameters, including the molecular weight and terminal functional group of PLGA polymers, and the emulsifying speeds for the formation of the oil-in-water emulsions were varied to observe the effect of the parameters on the release behavior of rivoceranib. The formulation parameters of the microspheres are summarized in Table 1. The fabrication parameters such as polymer end group did not significantly affect the efficiency of drug loading, suggesting there may be no interactions between the polymer end groups and rivoceranib.

**Figure 1:**
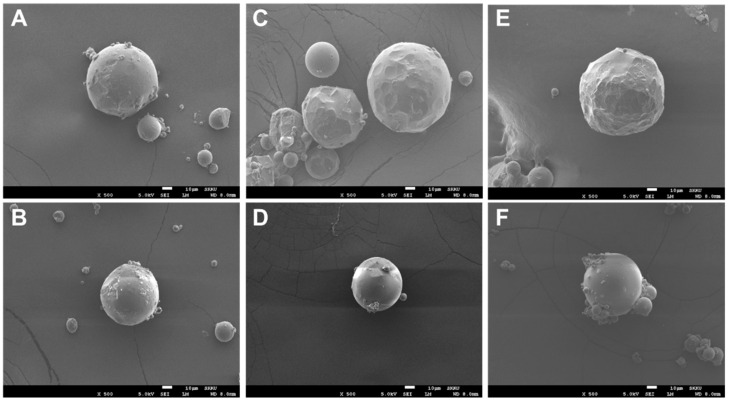
SEM images of rivoceranib microspheres. (**A**,**B**) microspheres were fabricated using PLGA RG502, (**C**,**D**) microspheres were from PLGA RG502H, (**E**,**F**) microspheres were from PLGA RG503H. Microspheres (**A**,**C**,**E**) were prepared at the emulsifying speed of 1500 rpm. Microspheres (**B**,**D**,**F**) were prepared at 2000 rpm. Scale bars = 10 μm.

**Figure 2:**
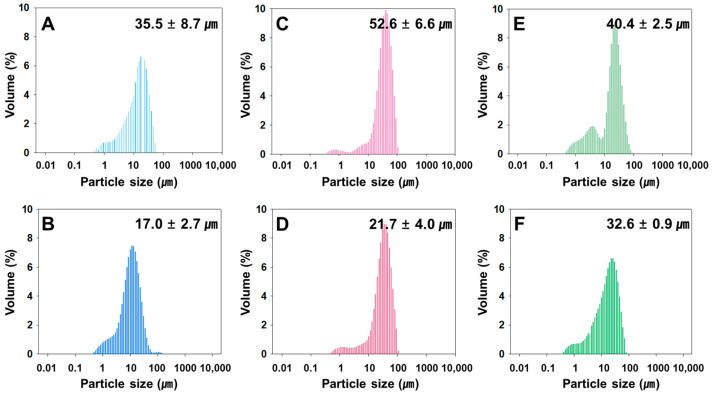
Size distribution of rivoceranib microspheres, determined by a light scattering method. (**A**,**B**) microspheres were fabricated using PLGA RG502, (**C**,**D**) microspheres were from PLGA RG502H, (**E**,**F**) microspheres were from PLGA RG503H. Microspheres (**A**,**C**,**E**) were prepared at the emulsifying speed of 1500 rpm. Microspheres (**B**,**D**,**F**) were prepared at 2000 rpm.

The rivoceranib microspheres showed a spherical morphology with a smooth surface, as observed in a scanning electron microscope (SEM) (Figure 1). The microspheres prepared at 2000 rpm of the emulsifying speed were smaller in size than those prepared at 1500 rpm (Figure 2). However, the viscosity of the polymer due to the difference in molecular weight seemed not to be significant.

### 3.2. Polymer–Drug Interactions

**Figure 3:**
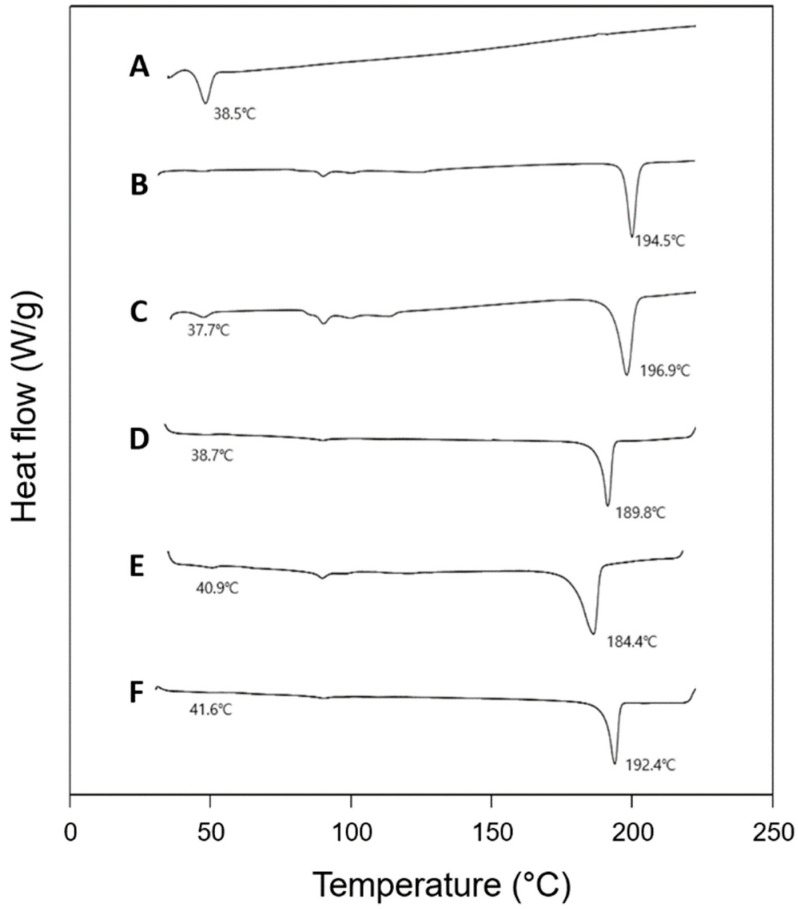
Differential scanning calorimetry (DSC) thermograms of (**A**) PLGA RG 502; (**B**) rivoceranib; (**C**) physical mixture (1:1) of PLGA RG502 and rivoceranib; (**D**) PLGA RG502 and rivoceranib (1:1) blend film; (**E**) PLGA RG502H and rivoceranib (1:1) blend film; (**F**) PLGA RG503H and rivoceranib (1:1) blend film. The downward peaks in the diagrams represent the endothermic behavior.

Interactions between a polymer matrix and a drug compound in a formulation are considered important since the interactions may affect the physicochemical properties and bioavailability of the drug [23]. The possibilities of polymer–drug interactions are often studied using the thermal and spectroscopic methods using DSC and FTIR, respectively [24]. The thermal behaviors of PLGA (RG502), rivoceranib, a physical mixture of PLGA and the drug (1:1, *w*/*w*), and polymer–drug blend films (1:1, *w*/*w*) are shown in Figure 3. PLGA RG502 and rivoceranib showed endothermic peaks at 38.5 °C and 194.5 °C, respectively (Figure 3A,B). It should be noted that the melting peak of rivoceranib appears in the polymer–drug physical mixture (Figure 3C) and the polymer–drug blend films (Figure 3D–F) with minor shifts in position, suggesting the absence of significant interactions that affect the crystalline nature of rivoceranib. The minor changes in the peak position may be due to the blending process, which decreases the purity of each component and, therefore, and do not indicate the polymer–drug interactions [25]. The effect of polymer–drug interactions on the thermal behaviors of the components was also observed in the rivoceranib-loaded microspheres. As shown in the blend films, no significant changes in the thermograms of the microspheres were observed, although the endothermic peak for rivoceranib was not detectable, possibly due to the low quantity of the drug (Figure S1).

**Figure 4:**
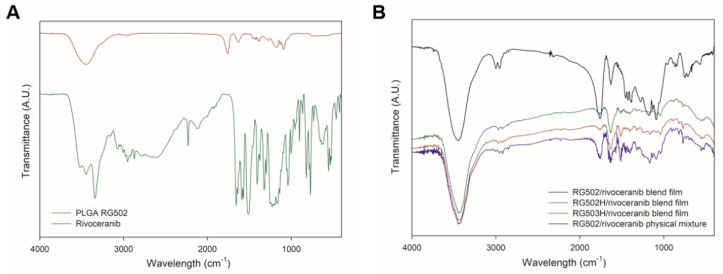
Fourier transform infrared (FTIR) spectrum of (**A**) PLGA RG502 polymer and rivoceranib; (**B**) PLGA RG502 and rivoceranib (1:1) blend film, PLGA RG502H and rivoceranib (1:1) blend film, PLGA RG503H and rivoceranib (1:1) blend film, and physical mixture (1:1) of PLGA RG502 and rivoceranib.

The potential polymer–drug interactions were further investigated using FTIR spectral analysis. The shifts of frequency and bandwidth of interacting groups indicate the changes in oscillation of the molecular dipoles in a polymer–drug mixture [26]. The characteristic peaks of PLGA (C=O at 1750–1705 cm^−1^; OH at 3500–3400 cm^−1^) and rivoceranib (-COOH at 1750–1700 cm^−1^; -NH at 700–600 cm^−1^; -C≡N at 2250–2200 cm^−1^) are shown in Figure 4A. The FTIR spectra of the polymer–drug physical blend films as well as the polymer–drug physical mixture did not show any absence of peaks of the functional groups in the spectra. In addition, no new bands were detected in the polymer–drug blend film. These results suggest that there are no significant chemical interactions, and the formation of new chemical linkages between PLGA and rivoceranib, demonstrating the significant compatibility between the two compounds.

### 3.3. Sustained Drug Release Profiles of Rivoceranib Microspheres

**Figure 5:**
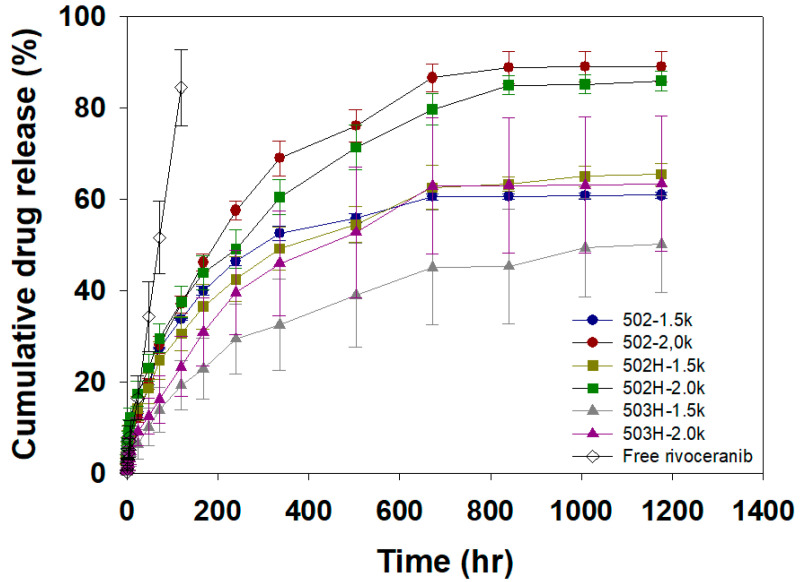
In vitro release curves of free rivoceranib and rivoceranib-loaded microspheres. The drug release profiles were represented as a mean ± SEM (*n* = 3).

In vitro rivoceranib release profiles from the rivoceranib microsphere formulations (Table 1) are shown in Figure 5. The release of free rivoceranib followed a zero-order kinetics with the cumulative drug release of 84% for 5 days. This result agrees with a previous observation with the release profile of a hydrophobic drug, paclitaxel, in an aqueous medium [27]. The microsphere formulations exhibited a burst release in a range from 1.4 to 5.7% over the initial 1 h. After the initial burst, the microsphere formulations achieved sustained drug release for 50 days. The relatively low initial burst and slow drug release profiles would be due to the hydrophobic nature of rivoceranib. The RG502−2.0k and RH502H−2.0k microspheres exhibited a similar drug release profile that resulted in 89.0 and 85.9% release during the experimental period, respectively. This indicated that the polar hydroxyl- and the ionizable carboxyl-end group of RG502 and RG502H did not significantly affect the drug release behaviors of the microspheres, suggesting the lack of significant interactions between the end group of the polymer chain and rivoceranib. The molecular weight of PLGA and the emulsification speed in the microsphere fabrication process significantly influence the release kinetics of the drug. The smaller microspheres prepared at a higher emulsification speed (2000 rpm), including 502−2.0k, 502H−2.0k, and 503H−2.0k, exhibited much faster drug release profiles than ones prepared at a lower speed, owing to the increased surface area [28].

### 3.4. Suppression of CNV Formation in Mice by Intravitreal Administration of Rivoceranib Microspheres

**Figure 6:**
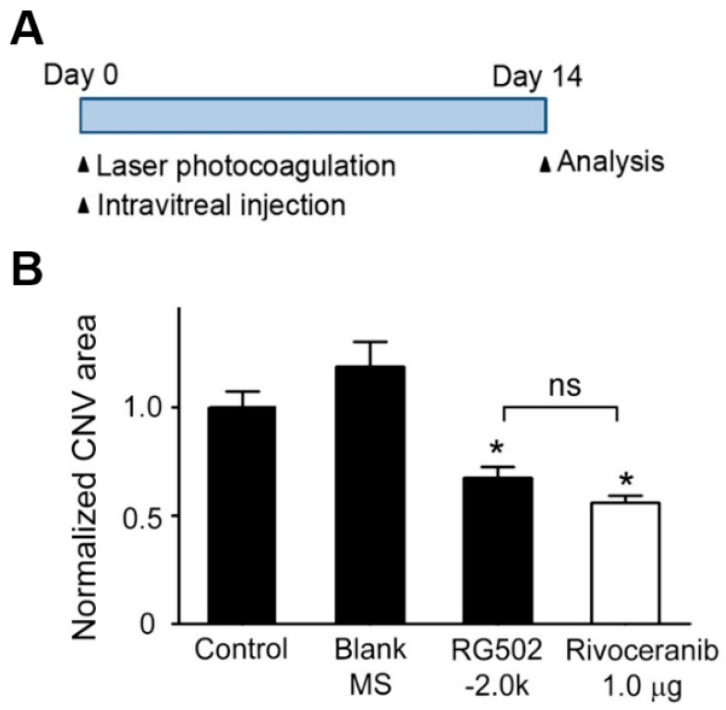
Inhibitory effect of rivoceranib-loaded PLGA microspheres and rivoceranib on laser-induced CNV formation in mice. (**A**) In vivo treatment schedule for the laser-induced choroidal neovascularization (CNV) experiment. (**B**) Immediately after laser photocoagulation, the mice received a single intravitreal injection of PBS (1 µL; *n* = 10 mice, Control), drug free RG502H microspheres (10% microsphere in 1 µL PBS, *n* = 4 mice, Blank MS), and rivoceranib-loaded PLGA 502H microspheres (10 µg rivoceranib/10% 502H−2.0k in 1 µL PBS, *n* = 10 mice). Two weeks later, the CNV area was analyzed. Rivoceranib (1.0 µg rivoceranib in 1 µL DMSO, *n* = 4 mice) and PBS (1 µL; *n* = 10 mice, Control). Two weeks later, the CNV area was analyzed. The CNV area was quantified by measuring the fluorescence intensity of images with isolectin IB4-positive areas. CNV areas of samples were expressed relative to that of control (one-way ANOVA with Bonferroni post hoc multiple comparison test, ns = not significant, * *p* < 0.05 vs. blank MS). Data are presented as the mean ± SEM.

To evaluate the ability of rivoceranib microspheres to suppress CNV formation in vivo, a mouse with laser-induced CNV was used. This model induced a rupture of Bruch’s membrane via laser photocoagulation, which causes the aberrant growth of new vessels from the choroid into the subretinal space [29]. The least effective dose of rivoceranib was determined as 1.0 μg, based on our previous results [30]. Immediately after laser photocoagulation, RG502H−2.0k microspheres containing 10 μg rivoceranib, RG502H−2.0k microspheres without the drug (blank microspheres), or PBS were intravitreally injected into mice. Two weeks later, the eyes were harvested and the area of CNV lesions was evaluated (Figure 6A). The posterior eyecups comprising retinal pigment epithelium (RPE), choroid, and sclera were flat-mounted and stained with isolectin B4 (IB4, stained in red). The CNV area was quantified by measuring the fluorescence intensity of images with isolectin B4-positive areas. Representative images of the IB4-stained CNV lesions show that the area of CNV lesions in RG502H−2.0k microsphere-treated mice was much smaller than those in PBS- or blank microsphere-treated controls (Figure 6B). Quantitative analysis showed that the area of CNV lesions in mice treated with RG502H−2.0k microspheres was 2.78 ± 0.21 × 10^3^ μm^2^, which was 2.12 ± 0.27 × 10^3^ μm^2^ and 1.35 ± 0.24 × 10^3^ μm^2^ lower than those in mice treated with blank microsphere and PBS, respectively. These results indicate that RG502H-2.0k microspheres efficiently block laser-induced pathological neovascularization in the choroid of mice.

## 4. Conclusions

In wet-type age-related macular degeneration (AMD), VEGF and its receptors have been recognized as primary disease targets, in which the increased expression of VEGF contributes to aberrant angiogenesis and vascular leakage, leading to vision impairment and loss. In this regard, we investigated the possibility of repurposing an anticancer drug, rivoceranib, as a therapeutic intervention for AMD, and the effect of biodegradable microsphere-based sustained delivery of the drug on pathological angiogenesis in the eye using a laser-induced CNV model that is a common animal model of wet-type AMD. The rivoceranib microsphere achieved sustained in vitro drug release for 50 days. The intravitreal administration of the microsphere effectively suppressed the abnormal blood vessel formation of the foveal avascular zone (subfoveal CNV). Considering the biocompatibility and clinical significance of PLGA microspheres, the rivoceranib microspheres could be considered as an economical and effective alternative to antiangiogenic antibody therapy for AMD.

## Figures and Tables

**Table 1:** Rivoceranib microsphere formulations.

Sample	Polymer Molecular Weight (Mw) ^a^	Polymer end Group	Emulsification Rate (rpm)	Drug Loading Content (%)	Encapsulation Efficiency (%)
502−1.5k	7000–17,000	Hydroxyl	1500	7.94	79.4
502−2.0k	7000–17,000	Hydroxyl	2000	7.38	73.8
502H−1.5k	7000–17,000	Carboxyl	1500	8.25	82.5
502H−2.0k	7000–17,000	Carboxyl	2000	7.89	78.9
503H−1.5k	24,000–38,000	Carboxyl	1500	7.80	78.0
503H−2.0k	24,000–38,000	Carboxyl	2000	8.77	87.7

^a^ as described in the manufacturer’s specification.

## Supplementary Materials

The following are available online at https://www.mdpi.com/article/10.3390/pharmaceutics13101548/s1, Figure S1: Differential scanning calorimetry (DSC) thermograms of (A) PLGA RG502 microsphere (drug free); (B) PLGA RG502/rivoceranib microsphere (502−2.0k); (C) PLGA RG502H microsphere (drug free); (D) PLGA RG502H/rivoceranib microsphere (502H−2.0k); (E) PLGA RG503H microsphere (drug free); (F) PLGA RG503H/rivoceranib microsphere (503H−2.0k). The downward peaks in the diagrams represent the endothermic behavior.
