# Supplementary material for: Sustained-Release Microspheres of Rivoceranib for the Treatment of Subfoveal Choroidal Neovascularization
Source: Pharmaceutics. 2021 Sep 24;13(10):1548. doi: 10.3390/pharmaceutics13101548 (PMC8538988; doi:10.3390/pharmaceutics13101548)
Supplement: Supplementary file 1 [file pharmaceutics-13-01548-s001.zip › pharmaceutics-1365781-supplementary.pdf]

pharmaceutics

MDPI

Supplementary Materials: Sustained-Release Microspheres of Rivoceranib for The Treatment of Subfoveal Choroidal Neovascularization

E Seul Kim, Min Sang Lee, Hayoung Jeong, Su Yeon Lim, Doha Kim, Dahwun Kim, Jaeback Jung, Siyan Lyu, Hee Joo Cho, Dong Min Kim, Wonhee Suh and Ji Hoon Jeong

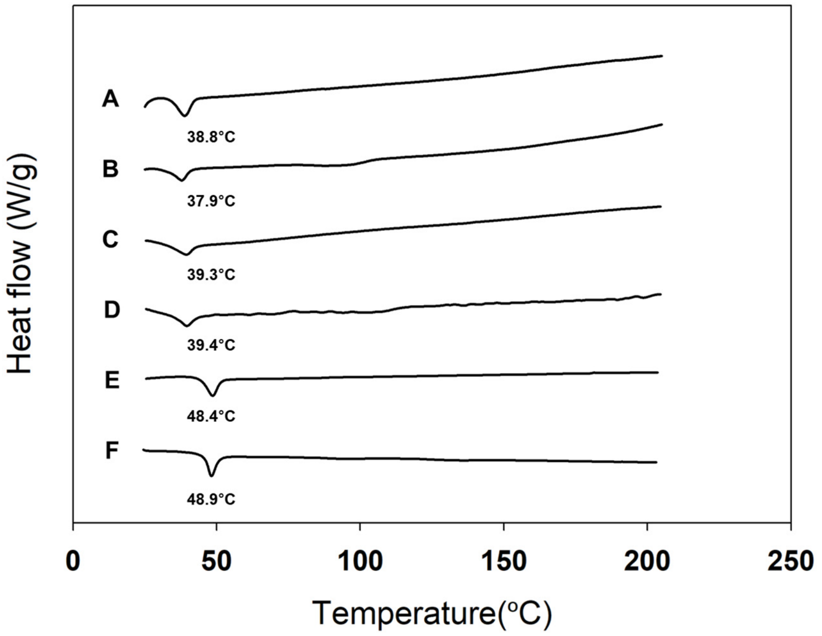

Figure S1. Differential scanning calorimetry (DSC) thermograms of (**A**) PLGA RG502 microsphere (drug free); (**B**) PLGA RG502/rivoceranib microsphere (502-2.0k); (**C**) PLGA RG502H microsphere (drug free); (**D**) PLGA RG502H/rivoceranib microsphere (502H-2.0k); (**E**) PLGA RG503H microsphere (drug free); (**F**) PLGA RG503H/rivoceranib microsphere (503H-2.0k). The downward peaks in the diagrams represent the endothermic behavior.
